# Lithium Use during Pregnancy and the Risk of Miscarriage

**DOI:** 10.3390/jcm9061819

**Published:** 2020-06-11

**Authors:** Eline M. P. Poels, Astrid M. Kamperman, Annabel Vreeker, Janneke Gilden, Marco P. Boks, René S. Kahn, Roel A. Ophoff, Veerle Bergink

**Affiliations:** 1 Department of Psychiatry, Erasmus University Medical Center, 3000 CA Rotterdam, The Netherlands; a.kamperman@erasmusmc.nl (A.M.K.); j.gilden@erasmusmc.nl (J.G.); r.ophoff@erasmusmc.nl (R.A.O.); veerle.bergink@mssm.edu (V.B.); 2 Department of Child and Adolescent Psychiatry/Psychology, Erasmus University Medical Center, 3000 CA Rotterdam, The Netherlands; a.vreeker@erasmusmc.nl; 3 Department of Psychiatry, Utrecht Medical Center, 3508 GA Utrecht, The Netherlands; M.P.M.Boks@umcutrecht.nl; 4 Department of Psychiatry, Icahn School of Medicine at Mount Sinai, New York, NY 10029, USA; rene.kahn@mssm.edu; 5 Department of obstetrics, gynecology and reproductive science, Icahn School of Medicine at Mount Sinai, New York, NY 10029, USA

**Keywords:** lithium, miscarriage, pregnancy, bipolar disorder, spontaneous abortion, perinatal, teratogenicity

## Abstract

Recent studies have provided new data on the teratogenicity of lithium. Less is known about the risk of miscarriage after lithium use during pregnancy. The aim of this study was to investigate the association between lithium use during pregnancy and miscarriage. Participants were women with bipolar I disorder and one or more pregnancies, of which information on medication use and pregnancy outcome was available (*n* = 443). The unadjusted odds ratios for miscarriage after lithium use during pregnancy was calculated. Multilevel logistic regression was used to calculate the odds ratio, adjusted for the age at conception and the clustering of pregnancies per woman. Miscarriages occurred in 20.8% of the lithium-exposed pregnancies (16/77), compared with 10.9% of the unexposed pregnancies (40/366) (OR = 2.14; 95% CI: 1.13–4.06). The adjusted odds ratio of miscarriage after lithium use during pregnancy was 2.94 (95% CI: 1.39–6.22). Lithium use during pregnancy may increase the risk of miscarriage.

## 1. Introduction

Women with bipolar disorder have a high risk of recurrent episodes in the perinatal period [1]. Treatment with mood-stabilizing medication during pregnancy might be necessary to reduce this risk, but this warrants special attention, as these medications may be potentially harmful to the developing fetus. Valproate and carbamazepine are highly teratogenic. Lithium has a well-established evidence base in the prevention of episodes in bipolar disorder [2] and is often prescribed during pregnancy, especially because, in 2012, a meta-analysis concluded that there was not enough evidence to say that lithium is teratogenic [3]. However, last year, the two largest studies to date were published, and they both showed the teratogenicity of lithium during the first trimester of pregnancy [4,5]. The first study compared 663 lithium-exposed pregnancies with 1945 lamotrigine-exposed pregnancies, and found a dose-dependent association between first trimester lithium exposure and cardiac malformations [4]. The second study reported an increased risk of major malformations (including cardiac malformations) in 727 first trimester lithium-exposed pregnancies, compared with 21,397 unexposed pregnancies in mothers with a mood disorder (OR = 1.62; 95% CI: 1.12–2.33) [5]. A few years earlier, a smaller study had found a similar, non-significant effect [6]. Interestingly, this study by Diav-Citrin et al. was the first to show an increased risk of miscarriage after first trimester lithium use (OR = 1.94; 95% CI: 1.08–3.48) [6]. In this prospective cohort study, 183 lithium-exposed pregnancies of women who had contacted the Israeli Teratology Information Service were followed up and compared with 72 disease-matched and 748 nonteratogenic-exposed pregnancies. Pregnancy outcome was assessed by maternal interview. The rate of miscarriage was 16.4% in lithium-exposed pregnancies, versus 8.3% in the bipolar disorder comparison group, and 5.7% in nonteratogenic-exposed pregnancies. In contrast, another prospective cohort study by Jacobson et al. did not find a difference in the rate of miscarriage between lithium-exposed and control pregnancies [7]. In this study, women were also recruited for study participation if they had contacted a teratogen information center, and pregnancy outcome was assessed by telephone interview. The rate of miscarriage was 9% in the lithium-exposed group (*n* = 138), versus 8% in a control group of women who used nonteratogenic drugs during pregnancy (*n* = 148). Other studies did not report on miscarriages, because they were designed to investigate live births only [8]. Information on the risk of miscarriage associated with lithium use during pregnancy is relevant for both clinicians and women with bipolar disorder of childbearing age. Based on the magnitude of this risk, further decisions regarding family planning and prevention strategies can be made. In this study, we present new information on miscarriages after lithium use.

## 2. Experimental Section

### 2.1. Study Design and Participants

This retrospective cohort study was part of the Dutch Bipolar Cohort (DBC) study, a collaboration between the University of California in Los Angeles and several Dutch healthcare institutes (University Medical Center Utrecht, Geestelijke gezondheidszorg (GGZ) Altrecht, GGZ inGeest, University Medical Center Groningen, Delta, Dimence, Parnassia (PsyQ) and Reinier van Arkel) [9]. Participants in the DBC study were patients with bipolar disorder, aged 18 years and older, between June 2011 and July 2015. The objective of the DBC study was to investigate the genetic and phenotypic information of the participants. The study was approved by the accredited Dutch Medical Ethical Trial Committee (METC 10-285) and all participants provided written informed consent [9]. In the current study, we selected a subcohort of women who had experienced one or more pregnancies, with a diagnosis of bipolar I disorder before pregnancy, and for which detailed data on lithium use and pregnancy outcomes were available. Analyses were performed on the total number of pregnancies that ended in live birth or miscarriage.

### 2.2. Data Collection and Procedures

For all patients, bipolar I disorder diagnosis was established using the Structured Clinical Interview (SCID) for the Diagnostic and Statistical Manual of Mental Disorders (DSM-IV), conducted by at least one well-trained independent rater [9]. In a self-report Questionnaire on Postpartum Mood Disorders, developed by one of the authors (V.B.), women were asked about the dates of the abortions, miscarriages, and births of their children. All participants were asked to complete a questionnaire, in which they were asked for detailed information on their current and lifetime medication use. In addition, they filled in a lithium satisfaction questionnaire, with specific questions on both their current and past use of lithium. Both questionnaires were combined to assess their current and past lithium use as accurately as possible and to restrain misclassification.

### 2.3. Statistical Analyses

Statistical analyses were performed using the Statistical Package for the Social Sciences (version 24.0, IBM Corp., Armonk, NY, USA). In order to investigate the association between lithium use during pregnancy and miscarriage, the odds of miscarriage were determined for pregnancies with and without lithium use. For our primary analysis, the unadjusted odds ratio for miscarriage after lithium use was calculated using a logistic regression model. Since an underlying maternal medical condition or genetic predisposition could cause multiple miscarriages within the same woman, this is a potential source of bias. In a sensitivity analysis, we adjusted for the occurrence of multiple miscarriages within one woman by means of a multilevel logistic regression analysis taking the clustering of pregnancies per woman into account. A generalized linear mixed model was defined with age at conception as a covariate in order to calculate the adjusted odds ratio. This generalized linear mixed model analysis was performed on a subgroup of pregnancies without comorbid lifetime valproate and carbamazepine use. Even though these medications are generally not prescribed during pregnancy in the Netherlands, they are teratogenic, and therefore we wanted to exclude any influence these medications might have had on the risk of miscarriage. Odds ratios were reported with their corresponding 95% confidence intervals. A two-sided *p*-value of 0.05 was considered to be statistically significant.

## 3. Results

In Table 1, we present the characteristics of our study sample. We analyzed the data of all the pregnancies of the women in the DBC study with a diagnosis of bipolar I disorder before pregnancy (*n* = 509), for which detailed data on lithium exposure and pregnancy outcomes were available (*n* = 443). Of these 443 pregnancies in 241 women, 56 ended in a miscarriage (12.6%; 56/443). The remaining pregnancies ended in a live birth (87.4%; 387/443). Lithium exposure varied over successive pregnancies. In total, 77 pregnancies were exposed to lithium (17.4%; 77/443) and 366 pregnancies were unexposed to lithium (82.6%; 366/443). Lifetime valproate or carbamazepine use was present in 26% of the lithium-exposed pregnancies and in 21% of the pregnancies not exposed to lithium. Miscarriages occurred in 20.8% of the lithium-exposed pregnancies (16/77), compared with 10.9% of the unexposed pregnancies (40/366), (OR = 2.14; 95% CI: 1.13–4.06, *p* = 0.018). After adjusting for the age at conception and the clustering of pregnancies per woman, the odds ratio of miscarriage after lithium use during pregnancy was 2.94 (95% CI: 1.39–6.22, *p* < 0.005).

## 4. Discussion

In the general population, miscarriage can be expected in 10–15% of pregnancies [10], which is similar to the rate of occurrence of miscarriage in our group of women with bipolar I disorder without lithium exposure. We found the rate of miscarriage to be increased in lithium-exposed pregnancies. This is consistent with data from the study by Diav-Citrin et al., who also reported the rate of miscarriage to be twice as high in lithium-exposed pregnancies (*n* = 183) when compared with disease-matched unexposed (*n* = 72) (OR = 1.94; 95% CI: 1.08–3.48) [6]. When we add the results of the current study to the previously published literature, we can conclude that two out of the three studies show an increased risk of miscarriage in lithium-exposed pregnancies [6,7]. This information warrants attention from clinicians treating women with bipolar I disorder of childbearing age. The risks associated with lithium use should be weighed against the risks of maternal recurrence. Maternal mood stability is also crucial for the wellbeing of mother and child, and the prevention of recurrence is especially important in women with a history of severe mood episodes. Lithium use during pregnancy lowers the risk of recurrence during pregnancy and postpartum for women with bipolar disorder [1,11], and lithium is less teratogenic than carbamazepine or valproate. Clearly, the risks and benefits of lithium use during pregnancy should always be weighed on an individual basis.

The association between lithium use during pregnancy and miscarriage in this study remained present after adjusting for the age at conception, the clustering of pregnancies per woman, and their lifetime use of valproate and carbamazepine. Importantly, the age at onset (an important indicator of the severity of illness in women with bipolar disorder) was similar in the lithium-exposed and unexposed groups, suggesting that the severity of illness does not explain the increase in miscarriages in the lithium-exposed group. Our results might, therefore, suggest a specific effect of lithium use during pregnancy.

The mechanism of the association between lithium use during pregnancy and miscarriage has not yet been investigated. We would like to propose a hypothetical mechanism of this association. Lithium use has been associated with overt and subclinical hypothyroidism in several studies [12] and (sub)clinical hypothyroidism has been associated with pregnancy loss [13]. Thyroid function might, therefore, have a mediating role in the association between lithium use during pregnancy and miscarriage. Unfortunately, thyroid levels during pregnancy were not available in this study. Further research is needed to study this hypothesis.

A few limitations need to be considered. In this study, data on pregnancy outcome and medication use were collected retrospectively by questionnaire and, therefore, recall bias might be present. However, a miscarriage is a major life event, and is likely to be remembered and reported by all women. Due to the fact that lithium use was assessed by questionnaire, we did not have information on lithium level and dosage during pregnancy and were not able to investigate a dose–response relationship. Additionally, information on maternal medical conditions, body mass index, smoking, alcohol and substance use at the time of miscarriage was not available and, therefore, it was not possible to investigate the potential mediating, moderating or confounding influence of these factors on our results.

## 5. Conclusions

Our findings suggest that, in addition or related to its teratogenic effect, lithium may increase the risk of miscarriage. These findings underscore the need for caution, but there is no imminent need to change clinical guidelines for lithium use during pregnancy, as current guidelines already warn against prescribing lithium during the first trimester of pregnancy. The possible risks associated with lithium use during pregnancy, such as the risk of miscarriage and congenital malformations, need to be carefully weighed against the risk of maternal recurrence. The current study provides important information that should be discussed with all women with bipolar disorder of childbearing age.

## Figures and Tables

**Table 1:** Characteristics of study sample.

	Total	Lithium-Exposed	Unexposed
*N* Pregnancies	443 in 241 women	77 in 50 women	366 in 202 women
*N* Miscarriages	56 in 41 women	16 in 11 women	40 in 30 women
Age at Conception, Mean (SD)	30.7 (4.9)	33.2 (4.6)	30.1 (4.9)
Age at Onset Bipolar Disorder, Mean (SD)	21.8 (6.3)	21.9 (5.1)	21.7 (6.5)
Lifetime Valproate or Carbamazepine Use *N* (%)	97 (21.8)	20 (26.0)	77 (21.0)
